# Adapting a Cancer Cost and Health Insurance Tool for Implementation: Rapid Qualitative Analysis

**DOI:** 10.1111/hex.70846

**Published:** 2026-08-31

**Authors:** Ashley J. Housten, Rachelle Roy, Krista Cooksey, Hannah Rice, Viktoria Vonder Haar, Sophia Larson, Su‐Hsin Chang, Jennifer Irvin, Mary C. Politi

**Affiliations:** ^1^ Division of Public Health Sciences Washington University School of Medicine St. Louis MO USA; ^2^ Program in Occupational Therapy Washington University in St. Louis St. Louis MO USA; ^3^ Bursky School of Public Health, Washington University in St. Louis St. Louis MO USA; ^4^ BJC HealthCare St. Louis MO USA

## Abstract

**Background:**

Cancer‐related financial hardships are common and negatively impact cancer‐related outcomes. Supporting patient‐clinician cost discussions, financial navigation, and insurance literacy may help mitigate this distress. This qualitative study informed the adaptation of the “Improving Cancer Patients’ Insurance Choices” (I Can PIC) tool into the “Cancer Affordability REsources” (CARE) Tool by updating content, design, and implementation processes.

**Methods:**

Patients ≥ 18 years, diagnosed with lung, prostate, colorectal, or gynaecological cancer within 3 years, English‐speaking, and treated at specified urban or rural cancer centres were included. Healthcare personnel (HP) were employed at one of these cancer centres and worked with the specified cancer types. The Consolidated Framework for Implementation Research (CFIR) and the 5As of Access in Healthcare Framework guided the development of semi‐structured interview guides and codebook. Interviews were conducted virtually, transcribed professionally, coded, and analysed using rapid thematic analysis.

**Results:**

Twenty‐two patients with cancer and 14 HP from urban and rural cancer centres participated (N = 36). We identified four themes. First, there was a mismatch of expectations around cost conversations. HP expressed concerns about potentially introducing financial‐related distress and did not know specific cost information, while patients emphasised the need for cost discussions with HP. One shared, “I'm kind of… removed from the financial aspect, which is good ‘cause then I can just focus on taking care of patients.” In contrast, one patient said, “…It's not acceptable to just have your hands up and say I don't know… a patient needs to know.” Second, preferences for timing of cost conversations varied, with a need for both immediate and subsequent conversations per patient readiness. One patient shared, “I would have loved to speak with someone about the cost, but I was more concerned about my survival…”. Third, there were multiple usability considerations, including technological demands, health literacy, and the perceived relevance of the tool. Fourth, context‐specific delivery is critical to ensure access.

**Discussion:**

This user‐centred content, usability, and implementation focused adaptation of the CARE Tool incorporated perspectives from patients, HP, and end‐users. Next steps will evaluate the CARE Tool in a clinical trial, assessing financial hardships, self‐efficacy, health insurance literacy, and implementation.

**Patient and Public Contribution:**

This research teams includes a patient advocate and our research question, design, data collection, and interpretation of results was presented for feedback to our community advisory board, made up of patient and public research partners. We recruited patients and healthcare personnel to participate in this study. We greatly appreciate the time, perspective, and experience that our patient and community research partners and participants have contributed to this study.

## 1 Background

Financial toxicity, the financial burden and stress related to cancer costs, is a common challenge for people with cancer and significantly impacts clinical outcomes and quality of life [1]. Financial toxicity is conceptualised across three domains: material hardship, psychological distress, and coping behaviours because of the costs of cancer care [2]. Financial toxicity affects people living in urban and rural settings as well as across various socioeconomic levels and insurance types [3, 4, 5]. Patients with cancer report their healthcare expenses can amount to 10‐20% of their annual income [6, 7]. To cope with financial toxicity, many patients diagnosed with cancer seek additional funds by identifying financial assistance sources, returning to work early, increasing borrowing and/or reduce spending via forgoing or delaying healthcare, choosing less expensive treatment options, or cutting back on non‐medical expenses [3]. However, not all patients diagnosed with cancer can make these financial modifications without substantial additional support.

Interventions designed to address financial toxicity employ multiple strategies such as enhancing patient‐healthcare professionals’ cost‐of‐care discussions, financial navigation, and insurance literacy training; these interventions can reduce or mitigate the financial distress accompanying a cancer diagnosis [8]. Some interventions support clinicians’ ability to address the cost burdens of their patients and can improve the quality of patient‐clinician cost conversations [9]. Others are delivered outside of clinical discussions and support patients’ understanding of cost‐related challenges. In a recent scoping review of interventions to address financial toxicity, 14% (n = 5) of 36 analysed studies included cost conversation prompts, 22% (n = 8) included direct financial/medical assistance, and 14% (n = 5) included financial counselling or coaching [10]; no dominant format was best and even low intensity interventions benefitted patients.

Formative work developed, implemented, and evaluated one such low‐intensity intervention: a web‐based tool called the Improving Cancer Patients’ Insurance Choices (I Can PIC). I Can PIC facilitated health insurance choice using plain language, graphics, and examples from survivors. An early version of the tool found those using the tool (vs. a control) had higher health insurance literacy, more confidence using insurance, and were more likely to choose insurance that matched their health and financial needs [11]. Subsequent modifications to the tool included cancer survivors’ input and cancer‐specific costs, which were evaluated in a randomised trial also demonstrating promising effects on health insurance knowledge and confidence making an insurance choice [12].

Although the I Can PIC tool showed promise in research settings with strong preliminary data and a user‐engaged development process, there were numerous challenges to sustaining implementation in routine clinical settings. In addition, I Can PIC was limited to those seeking coverage in the private insurance market; many who were uninsured or insured by government insurance programmes could also benefit from financial resources [12]. To address the implementation gap and update the tool with current cost needs in a changing health insurance landscape, we aimed to adapt I Can PIC to the Cancer Affordability REsources (CARE) Tool. This paper describes the process of soliciting feedback from end‐users to update the format and content of the tool, across multiple levels of influence, including at the patient, clinician, and organisation levels.

## 2 Methods

### 2.1 Design and Setting

The purpose of this study was to characterise how healthcare personnel (HP) and patients could envision using the tool to support patients navigating care costs and insurance after a cancer diagnosis. The goal was to sustain use in routine care. We used qualitative semi‐structured interviews to elicit feedback on the content and design of the I Can PIC website and advice for its implementation into routine practice. The qualitative interview guide was based on the Consolidated Framework for Implementation Research (CFIR) [13]. We built on our existing research partnerships with an urban comprehensive cancer centre and a rural cancer centre in Missouri to recruit HP and patients, allowing for diverse perspectives on the tool from two distinct geographic settings. The study was approved by the Washington University Institutional Review Board (IRB ID: 202411014).

### 2.2 Patient and Public Contribution

Early improvements and implementation insights for the CARE Tool prior to accrual for this research has been driven by input from our community co‐lead and patient advocate (JI) and members of the Community Advisory Board (CAB) of the larger parent grant, WashU‐ACCERT (U19CA291430). Our patient advocate, a staff member of our hospital partner for over 25 years, has assisted with the creation of patient‐facing materials for recruitment and facilitation of meetings with CAB members to elicit feedback on the CARE Tool. She continues to offer her perspective centreing the patient‐experience and social determinants of health alongside the CAB feedback.

### 2.3 Participants and Recruitment

To recruit patients, we posted information on social media platforms with cancer survivors, reviewed tumour registries, and asked healthcare professionals to refer eligible patients. Patients’ inclusion criteria were: 18 years of age and older, diagnosed with and/or treated for cancer within the previous 3‐years, able to read and write English, and received treatment at either the urban or rural cancer centre. Using purposive sampling methods, our aim was to recruit roughly equal numbers of patients per cancer type (i.e., gynaecological, prostate, lung, colorectal), per centre. Patients were contacted by a research coordinator after being identified in the tumour registry or after expressing interest in the study by completing a form via Research Electronic Data Capture (REDCap) [14] that was linked from a recruitment flyer. Interviews were scheduled with patients who were eligible based on inclusion criteria.

HPs who (1) were currently working at the rural or urban cancer centre and (2) had experience working with patients with lung, colorectal, prostate, or gynaecological cancer were recruited by sharing a flyers in person or via publicly available email addresses. HPs could have been members of the clinical care team (e.g., oncologist, nurse practitioner, nurse), or, due to the financial nature of the tool, an administrative staff member involved in patient billing. Interested HPs were also directed to REDCap to confirm eligibility and were subsequently contacted to schedule an interview.

### 2.4 Theoretical Frameworks

Through our qualitative investigation we sought to identify the multilevel pathways by which patient and HP factors, and the inner and outer settings of participating sites may influence implementation. We sought to understand these influences and how they may be interconnected to develop implementation strategies that are acceptable, appropriate, and able to be incorporated into routine care.

The Consolidated Framework for Implementation Research (CFIR) [13] provides a comprehensive list of constructs arranged across 5 domains that are likely to influence implementation. CFIR is a well‐recognised conceptual framework to guide systematic evaluations of multilevel factors that shape the effectiveness of intervention implementation. The CFIR domains as applied to this study are as follows: (1) Intervention Characteristics: characteristics of the CARE Tool, (2) Outer Setting: SDOH impact on uptake or implementation, (3) Inner Setting: compatibility of the CARE Tool with existing clinical workflows, (4) Characteristics of Individuals: knowledge and attitudes of end users, and (5) Implementation Process: end users’ reaction to implementation process proposed. Using a deductive approach, we employed CFIR to guide development of our semi‐structured interview guides and the qualitative code book.

The interview guide development also integrated the 5As of Access: affordability, availability, accessibility, accommodation, and acceptability [15] given the focus on care cost resources and support.

### 2.5 Interviews

Patient and HP interview guides were designed to elicit feedback on content and design of the website, followed by questions rooted in the CFIR constructs to collect insight for implementation of the tool in the context of cancer care. A qualitative researcher with over 10‐years of experience (AJH) oversaw the interview guide development. A team of two researchers (RR, VVH) who were trained in qualitative interviewing methods conducted the interviews. The interviews began with a review of consent information, a collection of participants’ verbal consent, and confirmation that the participant reviewed I Can PIC. Participants were also offered to view a screenshare of the tool at the beginning of the interview to aid in recall. All interviews concluded with a short demographic survey. The interviews were conducted individually over the video conferencing platform, Zoom, for up to 45 min. Participants were compensated with a $50 gift card for their time.

### 2.6 Data Analysis

Audio recordings from interviews were transcribed verbatim through a professional transcription service and the interviewers reviewed the transcripts prior to analysis to confirm de‐identification and accuracy. Transcripts were then uploaded to NVivo QSR 14 to begin the coding process. All interview guides and interview data are available via Qualitative Data Repository [16].

The coding team was made up of four investigators (RR, VVH, SL, and AJH). Three coders (RR, VVH, AJH) took part in an initial coding review session to test the first iteration of the rapid qualitative analysis template for coding an HP transcript [17]. After minor revisions to the master template for coding, the remaining transcripts were divided between a minimum of two team members (RR, VVH, SL) to summarise data within the template and transfer summaries into a matrix. The coding team met regularly to review independent coding, come to consensus, and discuss the application to the 5As of access and relevant CFIR constructs. If consensus could not be reached, the PI (AJH) made final decisions. Throughout the coding process, feedback on content and design were iteratively collected and categorised by feasibility for updating the website. Once all transcripts were coded and summarised in the matrix, the team identified suggestions for implementation and emerging themes that were coded from interviews and categorised them based on CFIR domains: Individual Characteristics, Implementation Process, Inner Setting, Outer Setting, and Innovation. An additional category, Delivery Options, was added to capture suggestions for how to optimise implementation from the perspectives of patients and HPs. The researchers referred to the original transcripts and excerpts throughout this process to ensure accurate reflection of the participants’ feedback. Rapid qualitative analysis was chosen for its accelerated timeline to ensure implementation needs and action‐oriented findings.

Once themes were generated, a two‐page document with sociodemographics of the study sample and our interpretation of the results was shared and presented with CAB members during their CAB meeting. CAB members offered their feedback and questions regarding these results, and suggestions were incorporated into the final analysis.

## 3 Results

Thirty‐six individuals completed interviews (Patients *n* = 22; HP *n* = 14). Tables 1 and 2 outline participant characteristics. Table 3 includes a summary of barriers and strategies to address these barriers. Below, we describe the themes identified within the data collected.

**Table 1:** Characteristics of patient participants.

Patients	
Total (*N*)	22
Care location:	
Urban centre	15
Rural centre	6
Both	1
Age: mean (SD)	52.7, (10.8)
Women (*N*)	11
Men (*N*)	11
Race/ethnicity:	
African American/Black	9
White	8
Hispanic/Latino	2
Another race or mixed race	3
Insurance policy holder:	
Yes	18
No (Spouse/partner of insurance policy holder)	4
Health insurance:	
Job or union sponsored	14
Medicare and/or Medicaid	6
Other	2
Cancer type:	
Lung	6
Colorectal	6
Prostate	5
Gynaecological	5
Relationship status:	
Married	12
Single	8
Widowed	1
Living with partner	1
Education:	
Some high school; High school or GED; Vocational training, associate's degree or trade, some college	12
Bachelor's degree or Graduate or professional degree	10
Household income:	
Less than $10,000	3
$10,000–$14,999	1
$15,000–$34,999	4
$35,000–$54,999	2
$55,000–$74,999	2
$75,000 or more	8
Prefer not to answer	2
Financial strain:	
There's not enough to make ends meet	5
There's enough to make ends meet, but not much for extras	10
There's plenty of money left over	4
Prefer not to answer	3
Mean health status^a^	3.5
Mean health literacy^b^	0.64

^a^ Scored from 1 – very good to 5 – poor.

^b^ Scored from 0 – extremely confident to 4 – not at all confident.

**Table 2:** Characteristics of healthcare personnel participants.

HP	
Total (*N*)	14
Care location:	
Urban centre	7
Rural centre	7
Women (*N*)	11
Men (*N*)	3
Race/ethnicity:	
White	13
Different race reported	1
Role:	
Ancillary services/physicians/Allied health professionals	12
Clinical/business operations	2
Years in role:	
< 5 years	5
≥ 5 years	9
Cancer type (worked with)^a^	
Colorectal	11
Gynaecological	11
Lung	10
Prostate	9

^a^ Not mutually exclusive, participants could select all of the cancer diagnoses they work with.

**Table 3:** Barriers identified by participants and strategies to address barriers.

Level	Barriers identified by participants	Strategies to address barriers
Healthcare Personnel (HP) level	Worry that cost conversations could introduce fear and tension. Mismatch between HP and patient expectations. Lack of time to discuss cost. Insufficient access to care cost information.	Train HPs on how to sensitively approach cost discussions. Train HPs on time‐efficient cost prompts and patients’ subtle cues about cost challenges. Share available and actionable financial resources with HPs to align with patient expectations.
Patient level	Potential overwhelm from an acute cancer diagnosis; patients need time to process diagnosis before discussing costs. Differing readiness and preferences for timing of cost conversations. Varying levels of technological skills and health literacy. Sensitivity around care costs and communicating financial hardship.	Introduce the tool in clinical flow 1–2 weeks after diagnosis. Revisit financial hardships and the tool throughout the post‐diagnosis cancer continuum. Improve usability and offer flexible, personalised, and multiple delivery options (e.g., non‐electronic modalities). Update usability, navigation, and design features in the CARE Tool to incorporate universal design and literacy best practices. Incorporate patient self‐efficacy strategies for discussing cost to reduce barriers, negative feelings, and experiences associated with cost discussions.
Organisational level	Unclear pathways for patient financial support and resources. Need for patient financial navigation.	Establish clear pathways in the CARE Tool to connect patients to financial resources when it is most preferred and needed for them. Identify organisational resources and financial navigation support.

### 3.1 Inner Setting: Discussing Care Costs May Lead to Mismatched Expectations

The challenges of discussing care costs can lead to mismatched expectations between patients and healthcare personnel. Patient participants reported that they would appreciate discussions about costs with their oncologists, trusting the information provided by them. As one patient shared,

… if somebody at the office would've said, “Hey, before your appointment in about a couple of weeks or whatever, you're gonna get an email from said website to help you with questions that you may have about insurance [that would help]. We always suggest that it's good to take a moment and go through that and if you have any questions, there'll be a number there for you to call ….

(PT08)

Patients wanted information from their HP to help direct and navigate them through all aspects of care, including costs. As one patient said,

I mean, the last time I asked the doctor, [laughter] they just came back with, “I don't know what the cost is gonna be, especially after the insurance, we don't know what the insurance is going to cover. And so I'm not sure what the final cost would be”. I think at that point, I just threw my hands up in the air and said, “Okay”. But I think, in hindsight, now, I would've said, “Well is there someone on staff that can help me with this?” Because… it's not acceptable to just have your hands up and say I don't know. Like, I need to know. Like, a patient needs to know.

(PT23)

Although it is important to patients to address costs, HPs felt tension between discussing care costs with patients without invoking assumptions or undermining trust. HPs do not always have cost information, even if they want to share it. Moreover, many HPs were conscious of the potential negative perceptions of asking about patient's ability to afford treatment. One HP mentioned,

I don't know your plan. I don't know all the details, and nor do I really wanna know that stuff. I think that, to me, I just wanna treat people and not segregate people you know… I wanna make my decision, like, ‘This is what you need, and then on the backside we're gonna work to get that for you no matter what.

(HP31)

Addressing financial concerns with patients early in the care pathway can be challenging for HPs, as they were cautious not to add to the patients’ overwhelm. For instance, a HP shared,

I feel like it would almost seem like pushy to be like and what's your insurance plan… they're already so overwhelmed with their cancer diagnosis that like I wouldn't want to task them with like making sure that their plan is the best plan at this very time.

(HP16)

Many HPs worried that addressing cost would imply patients might not be able to pay for their cancer care, and they preferred to defer these conversations until after care decisions were made.

HPs often lack information on specific treatment costs and clear pathways for connecting patients to financial assistance. They view cost conversations as outside of the primary role for the care they provide. One HP shared,

:… as a nurse I'm not educated in that … it's kind of out of my scope of practice. Like it's not something we as nurses like go through and learn about, so sometimes I feel like I'm just passing the buck on because I don't know an answer to it.

(HP06)

HPs reported barriers to including questions about their role in providing care costs in a clinical setting, their knowledge of care costs, knowledge of institutional resources (human and financial), and time. One HP stated,

… realistically, there's no time for myself to review this with patients just for how much we have to accomplish in that time… I think that the patient could review it on their own, and … probably reach out to … the numbers that you had given there for… financial assistance.

(HP16)

Another HP shared,

But as far as like a staff person setting aside time during their workday to individually go through the website with a patient, I think in a… clinical practice… usually people are busy with just the clinical care and they might not have time to go through a website.

(HP11).

However, when there were team members dedicated to financial navigation, HPs had a process within their clinic for connecting patients directly to those team members dedicated to financial navigation.

… I'm kind of a little bit removed from the financial aspect, which is good ‘cause then I can just focus on taking care of patients. And so usually, when patients have financial issues, it usually goes through other administrative people who, you know, through the billing office or something like that where they might refer patients. Like if a patient's having trouble paying their bills, then that office might refer them to be seen by the financial counsellor.

(HP11)

### 3.2 Individual Characteristics: Patients’ Ability to Balance Cost Discussions With the Emotional and Cognitive Demands of a New Diagnosis

Although patients wanted to talk about costs, and early in the care pathway, they still needed time to focus on their diagnosis upfront. For the majority of patient participants, their health and the overwhelm of being diagnosed with cancer was more important than how they were going to pay for their care upfront. Yet, waiting too long to address costs meant that a care pathway might already be chosen without attention to cost, which can impact ability to implement and pay for care. One patient shared,

But right now, when I was on this—on the diagnosis, I'm saying, I was really just wanted to get better. Okay? So, the bills and all that kinda stuff, it just… I didn't really care about the bills then…I just wanted to get well.

(PT12)

Another patient participant corroborated this sentiment:

… if it was available to me at the time, I would have loved to speak with someone about the cost, but I was more concerned about my survival and would have… done anything… the cost was irrelevant to me.

(PT14)

Patient participants shared that it was challenging to cope with both a cancer diagnosis and associated treatment costs simultaneously. Patients shared they could benefit from time to process their diagnosis before discussing care costs. For example, one patient described,

… getting a diagnosis like cancer is pretty overwhelming, and then someone to throw like, oh, money at you. Oh, well, no they just thinking about money; like I'm over here dealing with cancer here, you know, wrapping my head around it. So, I would say like probably giving them a little bit of time.

(PT15)

HPs observed the tension with timing too, highlighting the nuance of bringing costs up early but being responsive to patients processing their cancer diagnosis by offering opportunities to bringing costs up at multiple timepoints. As an HP stated,

… this is a difficult one for me. I just think it needs to be like mentioned, and then it needs to be reintroduced. Like mentioned again, not just like mentioned once… and then not mentioned again. But then once somebody has had like the proper time to kind of process everything, then it like be brought to them again…

(HP6)

Some HPs were more adamant about bringing up costs early because there are patients thinking about it.

… if they meet the doctor and… there's discussion about treatments and… then they may start thinking about that [costs] right away… for those patients, uh, who have that [cost] as a concern, I think it's always in the back of their mind… so, I think immediately would be good.

(HP11)

Introducing financial planning and a care cost and insurance tool early in the diagnosis process, but after initial conversations about diagnosis, can help prepare for future financial demands without burdening patients, by giving them time to process cost information. For example,

I think once I became aware of, you know, everything that was going on, I think if somebody had said, “Hey, here's a link to this. You know, go on there,” that would have kind of given me a little more peace of mind. Because that was one of the first things I worried about, you know, being that my husband's on a fixed income. You know, what if the cost of all of this is just going to be astronomical? You know, and I didn't have any idea, you know, of how to figure that or what to do.

(PT37)

… even though I do the budgeting for our family and stuff like that… healthcare is like one of those things that you don't always remember to budget in, so I guess like moving forward that is something… like revisiting this information is like, okay, that's probably something we should budget in for out‐of‐pocket costs… at that point in my life, my, I didn't really think about it [until] now.

(PT15)

### 3.3 Implementation Process: Adaptability Supports Context‐Specific Delivery

Context‐specific delivery is a priority for sustainability. While most participants shared that introducing the CARE Tool after an initial treatment plan is established and the patient has had time to process, there was not an overall consensus about timing. Introducing the CARE Tool to patients may require flexible timing of delivery and the opportunity to access it at different points. For example, one patient shared,

I think [other patients] would come back to it… If I'm not sure about stuff, I reuse it and try to go over it two or three times to make sure I'm getting the right information out of it.

(PT30)

Timing for delivery should be responsive to the patient and context, not solely about timing but how to initiate cost care conversations. As one clinician shared,

…it's not about when necessarily; it's just about how you present it and the wording… I think the earlier the better, you know? And if they don't wanna do it, they're not gonna do it. You can hit'em again 3 months down the road or 6 months down… hit'em again before open enrolment. I mean, that's really the time, right?

(HP31)

There is also a lack of consensus about the best mode of delivery which demonstrates a need to deliver the tool in a variety of ways that are personal and accessible to each individual patient. One HP shared,

… I think [patient portal] is great. I think that, you know, for the people who use it, I think that would be the best way… we have a handful of older adults who are really good at that, and then another handful… they don't even have a smartphone… it might even be used as a tool in an office, you know, like if you sat down and… I had to talk to someone about it in the office and they couldn't do it at home, I would pull it up and use it.

(HP04)

One patient participant stated that it may be hard for some without a smart phone to access the CARE Tool in clinic or at home, stating,

…. everybody's cell phone is pretty much a computer these days, you know? Scanning a QR code, I think it's very common these days, and it would help. However, I have a brother‐in‐law and it's a good thing he's a vet, but he still uses an old flipping flip phone, right? So he doesn't have that scan capability and all that, and he hates all of that stuff, right? So what do you do for those people ‘cause they still exist, right?

(PT08)

Some thought receiving the tool through the patient portal and/or electronically sent directly from the care team were described as the most desirable methods. As one patient participant said, “… I am the biggest fan of [patient portal], so if it came through [patient portal] with a link, that be effective. (PT07).”

### 3.4 Innovation: Usability Challenges of Web‐Based Cost Conversation Tools

Web‐based tools can support cost conversations outside the clinical encounter, but there are some concerns regarding usability barriers to delivery. For example, some patients preferred to have someone walk them through a website to ensure trust and access.

If there was [person] a that said, “This link might help you understand how how the insurance that you have is gonna play out with your treatment,” I guess I don't really know. What point do you know you're not in‐network? Or you're out‐of‐network? And at some point the insurance company tells you things, but I guess it would be good to know. It should come from the care team somehow.

(PT32)

When care costs start to become even more relevant as bills arrive, patient participants shared it would be ideal to reduce the burden for patient self‐advocacy by having a facilitator to introduce the web‐based tool in a trusted environment. Three participants described their perspective:

I think that telling people that there's a website is not going to be as beneficial as taking a minute or two to show them the website and kind of what's on it. Once they see it, I think they'd be like, “Oh, okay. I do need to learn more about that,” as opposed to just being like, “Oh, there's a website out there to help you understand insurance choices.

(PT17)

I need as much help as I possibly can, and I'm … more comfortable talkin’ with a person. It's cool to have a website as far as maybe screenin’ purposes, or even for those who might be a bit more tech‐savvy or who understand the lingo better. I just know me at [age], I want to talk to somebody named Roberta [laughing] on the phone.”

(PT21)

I do like the idea of it being coming from a real person versus a text or patient portal… especially when you're in that mode of you're seeking out healthcare and healthcare questions, you're already in an emotional and fragile state, so having a person reach out to you is fantastic.

(PT23)

Patient participants outlined potential barriers to using a website about costs include age and technological proficiency, financial literacy (including accessing health insurance information), health literacy and perceived relevancy. One patient participant mentioned help from family in accessing web‐based tools:

I mean, my daughter here, she's right here with me, and she has to walk me through everything'cause I don't do computers and stuff…I barely do a phone.

(PT33)

Another patient participant similarly described:

… I wish I could've seen it better, and as you can tell… I wear reading glasses, and… I use my cell phone a lot, so the layout's kind of important for me [so it's large enough to see].

(PT19)

HPs also shared their concerns with older adult patients using the CARE Tool. As one HP participant shared,

… I would probably tell you patients over 70 are probably gonna have difficulty just because they don't do well with the internet…I think the younger population, absolutely, um, older population, their kids might use it but most likely not [them].

(HP03)

Patient participants questioned the relevance of the cost calculator in the CARE Tool based on their existing insurance knowledge, whether bills have already start to come in (e.g., patients think this may be less applicable to them), and insurance type (Medicare and Medicaid *vs.* private insurance). A limitation of the CARE Tool is that it does not include cost data for Medicare or Medicaid plans, thus, one participant shared,

… [the cost calculator] wouldn't let me go any further with trying to fill the information in for the insurance I have. I mean, I put Medicare, I put Medicare Advantage … And it's when I would click next after entering all different kinds of information, it [says],“you qualify for Medicare”; I'm on Medicare already…

(PT36)

[About the cost calculator]: I just didn't fill all of that out… I didn't put in my age and any of that kind of stuff because I don't know, I didn't think that it was really for me specifically, right? … I've gone through whatever I've gone through, right? And I've seen our bills and I know what our insurance plan is and all that sort of thing. So it's not like there's anything that I need to choose, per se.

(PT08)

Organisational‐level barriers included limited clarity regarding the human and financial resources available to help patients navigate their care costs. As one HP shared,

You know, I think at [urban comprehensive cancer centre], you have so many different workflows for all the different clinics, right? Like, Head and Neck Surgery has a different workflow than Thoracic Surgery, and it's different from Rad‐Onc… you've got so many different things, and the navigators there aren't necessarily touching all the patients, right? And so that makes it more complicated, so you almost really have to have a champion in every department that's gonna try to figure out how it gets integrated to intake and then how you don't necessarily duplicate that for patients that are consuming multiple services, right?

(HP 31)

## 4 Discussion

This study explored patients’ and HPs’ perspectives on the implementation and usability of the CARE Tool, a health insurance and cost conversation tool for patients with cancer, to guide our user‐centred update of the tool. We identified several critical themes that offer insights into the expectations around cost conversations, the processing time needed for patients facing a cancer diagnosis, and multi‐level barriers for implementing the tool effectively.

Our results suggest a mismatch between patient expectations and clinician perceptions regarding cost discussions. HPs expressed a strong desire to avoid tension associated with asking about patients about financial concerns, often avoiding discussions about costs. This reticence, while intended to be sensitive, may overlook the needs of patients who are searching for guidance and transparency around care costs. This mismatch highlights the necessity of developing structured approaches to cost conversations that consider the sensitive nature of discussing costs, the capacity and knowledge of HPs, and patient expectations. These findings are supported by prior research outlining the need for multi‐level integration of cost conversations in routine clinical cancer care [12, 18, 19]. In addition, training opportunities for HP that include cost prompts, strategies to observe subtle cues from patients, and approaches to provide caring conversations to improve the quality of cost conversations among all patients are needed [9, 20]. Incorporating these multilevel intervention features that are both patient‐ and clinician‐centred may improve uptake and sustainability of cost conversations [18, 21].

Patients indicated a need for time to process their cancer diagnosis and explore their treatment and survival before they are ready to engage in discussions about care costs. Being responsive to patient needs during this time, highlighting the nuance of a new cancer diagnosis and associated care costs, was also supported by HPs. This finding highlights the importance of appropriately considering when and how frequently to introduce tools such as the CARE Tool. On the one hand, earlier financial planning could prevent hardship later in their treatment journey, but too soon could be overwhelming for patients as they coped with cancer and associated treatment information. Participants suggested a strategic introduction of the tool perhaps a week or two after diagnosis, with scheduled follow‐up engagements to revisit financial planning when patients are more prepared to process the information. Preparing for the reality of the financial demands of cancer care was a priority while allowing patients’ flexibility to select when they are ready to engage with cost information. Extant research supports the need for members of the care team, including oncologists, initiating cost conversations early in the cancer care pathway, but to assess the readiness of patients to discuss and revisit cost information [22, 23].

Opinions differed regarding the most opportune moments within the clinical pathway for implementation, including who should introduce the tool and how, emphasising a need for flexible and personalised delivery methods. Both patients and HPs suggested that the tool should be accessible at any time and integrated into patient portals or other electronic methods that fit into patients’ skills and routines. We suggest multiple delivery options to accommodate different patient preferences and technological capabilities.

There were several usability and delivery barriers to implementing the CARE Tool at the inner setting, outer setting and implementation process levels. Relevancy, technological proficiency, financial strain, as well as health literacy, were significant patient‐level barriers. Some patients—depending on their age and technological proficiency—may require facilitation when using the CARE Tool, However, HPs anticipated obstacles to their ability to provide this due to a lack of time and an insufficient access to information about care costs that may be required if questions about the Tool or about costs arise. Organisational‐level barriers included unclear pathways for patient financial assistance and limited resources dedicated to patient financial navigation. An additional limitation is this study was conducted in one state in the United States and restricted to those who identified as English‐speaking. To improve implementation transferability in the future, conducting research across geographically and linguistically diverse populations would advance this line of implementation research.

## 5 Conclusion

Addressing the multi‐level barriers identified through this qualitative enquiry may support future integration of the CARE Tool into clinical care. At the organisational level, our findings underscore the critical need for financial resources and established pathways to connect patients to them when needed. At the healthcare personnel level, knowledge of available financial resources and skills for engaging in cost conversations in ways that align with patient expectations is also needed. Training the various healthcare personnel involved in cancer care how to sensitively incorporate cost discussions into routine practice will be central to the implementation of the CARE Tool. Finally, at the patient level, the introduction of financial resources like the CARE Tool should be timed carefully, considering the patient's readiness to address financial concerns. In addition, improving the usability of the CARE Tool for patients with varying levels of technological proficiency and health literacy will be necessary.

Overall, this study provides formative evidence to guide implementation of financial support resources like the CARE Tool into routine cancer care. Findings highlight the importance of flexible timing, diverse delivery methods, and training for healthcare personnel to facilitate cost conversations. Ongoing research will evaluate the CARE Tool's impact on patient outcomes such as financial toxicity, self‐efficacy in cost communication, health insurance literacy, quality of life, and delayed or avoided care.

## Author Contributions

**Ashley J. Housten:** conceptualisation, project administration, writing – original draft, resources, supervision, funding acquisition, investigation, writing – review and editing. **Rachelle Roy:** investigation, writing – review and editing, project administration, formal analysis. **Krista Cooksey:** investigation, writing – review and editing, project administration, formal analysis. **Hannah Rice:** investigation, writing – review and editing, formal analysis. **Viktoria Vonder Haar:** investigation, writing – review and editing, formal analysis. **Sophia Larson:** investigation, writing – review and editing, formal analysis. **Su‐Hsin Chang:** conceptualisation, writing – review and editing, funding acquisition. **Jennifer Irvin:** conceptualisation, writing – review and editing, funding acquisition. **Mary C. Politi:** conceptualisation, writing – review and editing, funding acquisition, supervision, investigation.

## Conflicts of Interest

The authors declare no conflicts of interest.

## Data Availability

The data that support the findings of this study are openly available in QDR at Syracuse University at https://data.qdr.syr.edu, reference number 5064/F6SXZFNA. Data from this study are available through the Qualitative Data Repository at Syracuse University (https://data.qdr.syr.edu/dataset.xhtml?persistentId=doi:10.5064/F6SXZFNA).
